# Diabetes incidence and projections from prevalence surveys in Fiji

**DOI:** 10.1186/s12963-016-0114-0

**Published:** 2016-11-25

**Authors:** Stephen Morrell, Sophia Lin, Isimeli Tukana, Christine Linhart, Richard Taylor, Penina Vatucawaqa, Dianna J Magliano, Paul Zimmet

**Affiliations:** 1 School of Public Health and Community Medicine, Samuels Building, University of New South Wales, Randwick, NSW 2052 Australia; 2 Ministry of Health and Medical Services, Dinem House, 88 Amy Street, Toorak, Suva, Fiji Islands; 3 National Food and Nutrition Centre, 1 Clarke Street, Suva, Fiji Islands; 4 Baker IDI Heart & Diabetes Institute, 75 Commercial Road, Melbourne, VIC 3004 Australia

**Keywords:** Developing country, Fiji, Incidence, Obesity, Pacific islands, Prevalence, Trends, Type 2 diabetes mellitus

## Abstract

**Background:**

Type 2 diabetes mellitus (T2DM) incidence is traditionally derived from cohort studies that are not always feasible, representative, or available. The present study estimates T2DM incidence in Fijian adults from T2DM prevalence estimates assembled from surveys of 25–64 year old adults conducted over 30 years (*n* = 14,288).

**Methods:**

T2DM prevalence by five-year age group from five population-based risk factor surveys conducted over 1980–2011 were variously adjusted for urban-rural residency, ethnicity, and sex to previous censuses (1976, 1986, 1996, 2009) to improve representativeness. Prevalence estimates were then used to calculate T2DM incidence based on birth cohorts from the age-period (Lexis) matrix following the Styblo technique, first used to estimate annual risk of tuberculosis infection (incidence) from sequential Mantoux population surveys. Poisson regression of year, age, sex, and ethnicity strata (*n* = 160) was used to develop projections of T2DM prevalence and incidence to 2020 based on various scenarios of population weight measured by body mass index (BMI) change.

**Results:**

T2DM prevalence and annual incidence increased in Fiji over 1980–2011. Prevalence was higher in Indians and men than i-Taukei and women. Incidence was higher in Indians and women. From regression analyses, absolute reductions of 2.6 to 5.1% in T2DM prevalence (13–26% lower), and 0.5–0.9 per 1000 person-years in incidence (8–14% lower), could be expected in 2020 in adults if mean population weight could be reduced by 1–4 kg, compared to the current period trend in weight gain.

**Conclusions:**

This is the first application of the Styblo technique to calculate T2DM incidence from population-based prevalence surveys over time. Reductions in population BMI are predicted to reduce T2DM incidence and prevalence in Fiji among adults aged 25–64 years.

**Electronic supplementary material:**

The online version of this article (doi:10.1186/s12963-016-0114-0) contains supplementary material, which is available to authorized users.

## Background

As incidence is a measure of new cases of disease occurring in a given time, it is preferable to using prevalence for analyzing risk factors and causality, and for projections. Incidence of type 2 diabetes mellitus (T2DM) is normally derived from cohort studies based on population samples, such as in Mauritius [1]. However, there may be limitations of the generalizability of incidence data to the rest of population from such cohort studies if they are not representative; and Hawthorne effects [2] might occur for some risk factor variables from repeated follow-up of the same subjects. Incidence of T2DM has also been calculated by self-report of new doctor-diagnosed T2DM in a defined retrospective period (such as 12 months) from cross-sectional surveys [3], as in the 1997 and 2003 United States National Health Interview Surveys. However, this method does not include those with undiagnosed hyperglycemia. New T2DM cases prescribed pharmaceutical treatments may be measured by notification through prescriptions, pharmacies, or reimbursement schemes in defined populations [4–6]. However, such registries may be incomplete and cannot identify those not prescribed medication for T2DM. In Denmark, a secular analysis of the national database of people with T2DM receiving medication found that prevalence increased due to reductions in mortality (increased survival), not due to increases in new registrations [6]. Incidence may be back-calculated using compartment models, as occurs with the WHO DisMod program [7], but this requires cause-specific mortality data which often are not complete in low-resource countries.

T2DM incidence from cohort studies in the Pacific Islands is limited to Samoa [8] and American Samoa [8]. Incidence of T2DM-related morbidity was measured in Nauru and showed a decline from 26.2 per 1000 person-years over 1975/76–1982 to 22.5 per 1000 person-years over 1982–87 [9]. In Samoa, the 4-year T2DM incidence over 1991–1995 was reported as 1% (29–43 years) and 4% (44–60 years) in each sex, equivalent to annual incidences of 2.5 per 1000 person-years (29–43 years) and 10 per 1000 person-years (44–60 years) based on the mean for the four-year period. When age-adjusted to the 1991 Samoa population census [10], this is equivalent to an annual T2DM incidence of 5.3 per 1,000 person-years for each sex. In American Samoa for the same age group, the average annual T2DM incidence (age-adjusted to 1990 American Samoa census) [11] was 28.7 per 1000 person-years (men) and 21.7 per 1000 person-years (women) over 1990–1994. In Fiji no prospective cohort study of T2DM has been conducted, but prevalence surveys have been performed over the last three decades. Analysis of T2DM prevalence trends based on six population surveys in Fiji indicates an increase from 7.7 to 15.6% between 1980 and 2011, with projection to 2020 estimated to be 19.3% [12]. Fiji’s population, approximately 828,000 at the most recent 2007 census, comprises 57% Melanesian (i-Taukei) and 38% Indian. The remaining 5% is heterogeneous and is composed of Asian, European, and other Pacific island populations [13].

It is possible to determine disease incidence from consecutive prevalence surveys from sequential samples in a population if the surveys are stratified by age and utilize similar methodology. This was demonstrated in the 1960s using recurrent prevalence surveys of tuberculosis infection, ascertained through Mantoux (tuberculin) skin tests, to determine the annual (incidence) rate of infection using birth cohorts from an age-period (Lexis) matrix [14]. No publications have been located using this technique to calculate (aggregate) incidence rates from prevalence surveys for T2DM.

In the present study, population-based prevalence surveys of T2DM and obesity conducted between 1980 and 2011 of Fijian adults aged 25–64 years are used to calculate T2DM incidence. T2DM prevalence and incidence are projected to 2020 using body mass index (BMI) as a predictor variable under different scenarios of population obesity.

## Methods

### Survey selection

Unit records from five non-communicable disease (NCD) risk factor population surveys were included for analysis (*n* = 14,288): the 1980 National Cardiovascular Disease and Diabetes Survey (NCVDS) [15], 1993 National Nutrition Survey (NNS) [16], 2002 Fiji STEPS [17], 2004 NNS [18], and 2011 Fiji STEPS [Draft WHO STEPS report]. Eight other cross-sectional health surveys conducted in Fiji since 1952 [19–26] could not be included in this analysis as unit records were unavailable, and published tabulated data could not be disaggregated into urban-rural, ethnicity, sex and five-year age groups. Consequently, these could not be made nationally representative. Only adults aged 25–64 years who were of i-Taukei (Fiji Melanesian) or Asian Indian descent and had a confirmed fasting status for fasting plasma glucose (FPG) were included. Those of “Other” ethnicity (<5%) were excluded due to the heterogeneity of this group, along with pregnant women where gestational diabetes can artifactually inflate the prevalence of T2DM.

### T2DM prevalence

In the 1980 NCVDS, glucose was measured in plasma from venous blood; for the 2011 STEPS, glucose was measured in capillary whole blood, but reported calibrated to a plasma concentration. T2DM was defined as FPG ≥7.0 mmol/L and/or on medication for T2DM [27]. Both the 1993 and 2004 NNS measured T2DM prevalence using self-report. In order to adjust to T2DM diagnosed including blood samples, logistic regression models were derived from the 2002 STEPS survey to adjust for under-enumeration of T2DM from self-report only data. In each sex-specific model, T2DM (FBG ≥6.1 mmol/L [whole blood glucose] and/or on medication) was modeled with ethnicity, age, BMI, and self-reported doctor-diagnosed T2DM status and/or on T2DM medication (for 2004 NNS), or self-reported, doctor-diagnosed T2DM status only (for 1993 NNS). Further details on the methods used to adjust self-reported T2DM data have been published previously [12].

For each survey, the five-year prevalence for each ethnic-specific sex group, and nationally, was calculated after being variously case weighted for urban-rural, ethnicity, and sex to the nearest previous census to minimize selection bias and improve representativeness at the time of each survey. Case weights were derived by dividing the proportion of the census sub-group by the equivalent proportion in the survey sub-group. Age-specific data were then age-standardized to the nearest previous census. Confidence intervals were calculated using binomial methods [28]. Adjusted T2DM prevalence counts for each survey year were then derived by multiplying the adjusted prevalence by the survey population for each sub-group.

### T2DM incidence

Annual T2DM incidence was estimated by adapting the method developed by Styblo et al. [14] to estimate tuberculosis incidence from prevalence of tuberculosis infection as assessed by sequential surveys of Mantoux positivity. The method commences with prevalence estimates of a disease for a given age and period, and hence year of birth. The mean annual probability of *not* acquiring the disease, from year of birth to a given age, for a given observation year, is then estimated from this prevalence. For each given age, a straight line is then fitted through a mathematically convenient transformation of this mean probability as a function of the year of observation. The regression β-estimate for this trend is used in conjunction with the observed prevalence for the given year and age group to estimate the cumulated probability of not having the condition for that age and year (according to formula (3) in Additional file 1: Supplement S1). One minus this quantity becomes the incidence estimate for the given age group and year. The details of this procedure are provided in Additional file 1: Supplement S1.

As the intervals between the surveys used for this study were not evenly spaced, age-specific T2DM prevalences were interpolated between the survey years to produce estimates of annual age-specific prevalences of T2DM for each sex and ethnic group, and the above procedure was applied to each interpolated prevalence.

Annual age-specific incidence estimates for each sex and ethnic group were then directly age-standardized to the 2007 Fiji census 25–64 year population separately by sex and ethnicity, and also nationally, to produce five incidence trends for 1980–2011 for ages 25–64 years. Estimated age-specific incidence projected to 2020, based on T2DM prevalence projected to 2020, was also directly age-standardized (2007 Census).

Confidence intervals (95%) are based on the normal approximation to the binomial using the survey populations for each survey year. For years between surveys, interpolated populations were calculated using the adjacent surveys. Counts of incident cases of T2DM for each survey year were derived by multiplying the estimated incidence rates by the survey population for each sub-group. The cumulative incidence risk (%) was calculated as [1-exp(-x)]*100, where *x* is the cumulative incidence rate, derived from the sum of age-specific incidence rates (*r*) times each age interval (*n*); i.e., Ʃ r*n [29].

### Projection of T2DM prevalence and incidence based on obesity scenarios

Projections of T2DM prevalence in Fiji have been calculated based on period trends [13]. In this analysis, the estimated annual counts of T2DM prevalence and incidence are modeled by Poisson regression against survey year (*n* = 5), ethnicity (*n* = 2), sex (*n* = 2), five-year age group (*n* = 8), and mean BMI for each of the resulting 160 strata. Mean BMI values for 2020 were derived from a random-effects linear meta-regression of six surveys [13] and used in the model to produce T2DM prevalence and incidence projection estimates for 2020 from models that incorporate BMI.

BMI-based projections of T2DM prevalence and incidence in 2020 are then re-estimated according to several population weight change scenarios, expressed as BMI: (1) mean population weight continues to increase at the current rate – predicted 2020 mean BMI from a linear period trend [13]; (2) mean population weight is maintained at 2011 levels (i.e., no weight or BMI gain); and (3) mean population weight is reduced or increased by 1–4 kg (with consequent changes in BMI) compared to 2011 levels, 2011 being the most recent year for which nationally representative empirical population survey data are available. A 1–4 kg weight change was selected as this represents a feasible 0–5% body mass change. Projected T2DM incidence rates in 2020 based only on period trends are compared to projected incidence rates for the same year from Poisson regression models including mean BMI for various obesity scenarios.

Data were analysed using SAS 9.4 [SAS Institute Inc., Cary, NC, USA], SPSS 22 [IBM Corp., Armonk, NY, USA], and Microsoft Excel [Microsoft, Redmond, WA, USA].

## Results

### T2DM prevalence

**Fig. 1:**
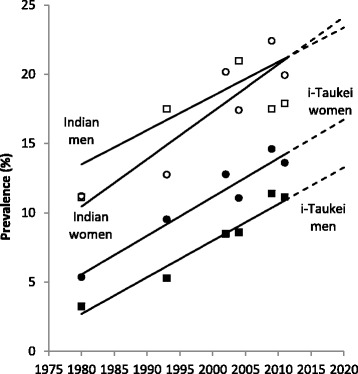
Diabetes (T2DM) prevalence (%) in Fiji adults aged 25–64 years, 1980–2011. *Black squares* = i-Taukei men; *black circles* = i-Taukei women; *white squares* = Indian men; *white circles* = Indian women. T2DM prevalence was adjusted for urban-rural and age to nearest previous census. *Trend lines* were derived from meta-regression of adjusted survey prevalences weighted by the inverse of the SE

T2DM prevalence increased with period and age, both nationally and in all ethnic-specific sex groups (Table 1, Fig. 1, Additional file 1: Supplement S2). Prevalence is higher in Indians than i-Taukei (Fiji Melanesian), and higher in i-Taukei women than i-Taukei men. In Indians, this is reversed: prevalence in men is higher than in women. Prevalence was highest in the oldest age groups and lowest in the youngest age groups.

**Table 1:** Diabetes (T2DM) prevalence (%) for Fiji adults aged 25–64 years in each survey year

	1980 NCVDS	1993 NNS	2002 STEPS	2004 NNS	2011 STEPS
25–29	1.8	3.7	5.8	4.6	3.6
30–34	4.9	5.6	5.3	5.9	9.0
35–39	5.5	8.4	9.5	8.5	6.7
40–44	7.2	11.0	15.5	12.3	18.7
45–49	11.3	17.2	20.2	20.0	20.0
50–54	18.3	19.9	31.9	24.3	26.7
55–59	15.5	24.5	36.3	33.0	29.0
60–64	17.0	26.5	36.2	35.6	36.8
**25–64** ^**a**^	**7.7**	**11.1**	**16.5**	**14.5**	**15.6**
95% CI	7.4–8.0	10.7–11.6	15.9–17.1	14.0–14.9	15.1–16.2

^a^Age-standardized to nearest previous census

Projected national T2DM prevalence in 2020 based only on period trends is 19.3% (Table 2), and the projection based on period and linear increases in BMI is 20.6%. National T2DM prevalence is projected to reach 18.8% in 2020 if mean population weight is maintained at 2011 levels. For a mean population weight reduction of 1–4 kg from 2011 levels, T2DM prevalence is projected as 17.9–15.5%. On average, for every 1-kg decrease in mean population body weight there is a 0.8% reduction in T2DM prevalence. When mean population weight is increased by 1–4 kg from 2011 levels, projected T2DM prevalence in 2020 is 19.8–23.0%. On average, for every 1-kg increase in mean population body weight there is a 1.1% increase in T2DM prevalence.

**Table 2:** T2DM prevalence (%) projections to 2020 based on various body mass index (BMI) scenarios, 2011–2020

	T2DM prevalence (%) and 95% CI				
	i-Taukei		Indian		Fiji
	Men	Women	Men	Women	
2011 STEPS prevalence^a^	11.13 (10.34–11.92)	13.61 (12.73–14.48)	17.90 (16.41–19.39)	19.93 (18.61–21.26)	15.61 (15.06–16.16)
Projected to 2020 from period trend only^b^	13.27 (13.22–13.33)	16.73 (16.66–16.81)	23.37 (23.09–23.65)	24.14 (23.99–24.29)	19.28 (19.19–19.37)
Projected to 2020 from period and BMI trends^c^	15.35 (12.12–18.58)	19.90 (16.83–22.98)	21.14 (16.88–25.40)	23.09 (19.37–26.81)	20.56 (18.78–22.35)
Projected: no weight change 2011-20^c^	15.18 (11.97–18.40)	18.16 (15.20–21.13)	20.67 (16.44–24.89)	21.73 (18.09–25.38)	18.82 (17.10–20.55)
Projected from mean weight change to 2020					
Weight loss 2011–2020^c^					
−1 kg	14.67 (11.50–17.84)	17.59 (14.66–20.52)	20.24 (16.05–24.43)	21.08 (17.47–24.68)	17.94 (16.25–19.63)
−2 kg	14.24 (11.11–17.37)	17.01 (14.11–19.90)	19.74 (15.59–23.89)	20.45 (16.89–24.02)	17.08 (15.42–18.74)
−3 kg	13.82 (10.73–16.92)	16.44 (13.59–19.30)	19.25 (15.13–23.36)	19.85 (16.32–23.37)	16.25 (14.63)
−4 kg	13.42 (10.73–16.92)	15.90 (13.09–18.72)	18.77 (14.70–22.84)	19.26 (15.77–22.74)	15.47 (13.87–17.07)
Weight gain 2011–2020^c^					
+1 kg	15.57 (12.32–18.82)	18.81 (15.80–21.82)	21.29 (17.02–25.56)	22.38 (20.85–23.92)	19.80 (18.04–21.56)
+2 kg	16.04 (12.75–19.33)	19.46 (16.41–22.51)	21.83 (17.52–26.14)	23.07 (19.35–26.79)	20.80 (19.01–22.60)
+3 kg	16.52 (13.20–19.85)	20.12 (17.04–23.21)	22.38 (18.04–26.73)	23.77 (20.01–27.53)	21.86 (20.03–23.68)
+4 kg	17.02 (13.66–20.39)	20.81 (17.69–23.94)	22.95 (18.57–27.34)	24.50 (20.70–28.30)	22.96 (21.11–24.82)

^a^Adjusted variously for urban-rural, ethnicity, sex, and age to 2007 census [13]

^b^From linear meta-regression of T2DM prevalence estimates from six surveys, age-adjusted [13]

^c^From ethnic-specific Poisson models using survey year, sex, age, and mean BMI

### T2DM incidence

**Fig. 2:**
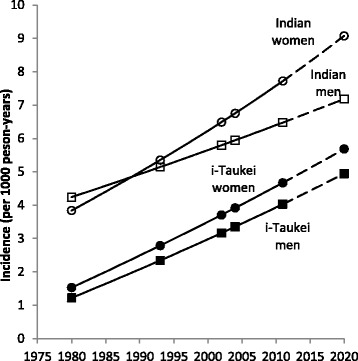
Estimated T2DM incidence (per 1,000 person-years) in Fiji adults aged 25–64 years, 1980–2010, with projection to 2020. *Black squares* = i-Taukei men; *black circles* = i-Taukei women; *white squares* = Indian men; *white circles* = Indian women. *Solid line* = interpolated and modeled incidence using the Styblo birth cohort method. *Broken line* = projected incidence

Annual national age-standardized T2DM incidence is estimated to have increased from 2.6 to 5.0 per 1000 person-years between 1980 and 2011 (Table 3, Fig. 2, Additional file 1: Supplement S2). The incidence rate increase has remained similar between the sexes in i-Taukei. In Indians, incidence is rising faster in women than in men, and has been higher than in men since 1990. Overall, between ages 25 and 64 years, the estimated cumulative risk of acquiring T2DM increased from 22% in 1980 to 37% in 2010, and by 2020 is projected to be 42%.

**Table 3:** Estimated annual T2DM incidence (per 1,000 person-years) and cumulative risk for Fiji adults aged 25-64 years for each survey year, 1980–2011

	1980	1993	2002	2004	2011
25–29	1.07	1.54	1.86	1.94	2.19
30–34	1.54	2.02	2.35	2.43	2.69
35–39	1.99	2.24	2.41	2.45	2.59
40–44	2.20	3.71	4.81	5.05	5.94
45–49	3.44	4.63	5.50	5.69	6.38
50–54	4.62	5.88	6.80	7.00	7.75
55–59	4.68	6.90	8.57	8.95	10.35
60–64	4.78	6.48	7.74	8.18	9.08
**25–64** ^**a**^	**2.61**	**3.58**	**4.28**	**4.45**	**5.02**
95% CI	2.52–2.69	3.30–3.86	4.04–4.53	4.19–4.70	4.72–5.31
Cumul risk (%) 25–64	21.60	28.40	32.99	34.09	37.48
95% CI	19.79–23.40	26.30–30.50	31.04–34.94	32.44–35.74	35.46–39.49

Incidence calculated by the Styblo birth cohort method [14]

^a^Age-standardized to nearest previous censuses

National annual T2DM incidence based on current-period BMI increases is projected to be 6.4 per 1000 person-years in 2020 (Table 4), higher than projected incidence based on period alone (5.8 per 1000 person-years). Projected ethnic- and sex-specific incidence rates in 2020 are also higher using BMI in Poisson regression models than in models with period alone (Table 4): i-Taukei men: 5.6 compared to 5.0 per 1000 person-years; i-Taukei women: 6.8 compared to 5.7 per 1000 person-years; Indian men: 8.0 compared to 7.2 per 1000 person-years; Indian women: 9.3 compared to 9.1 per 1000 person-years.

**Table 4:** Annual T2DM incidence (per 1000 person-years) projection to 2020 based on various BMI scenarios, 2011–2020

	T2DM incidence (per 1,000 person years) and 95% CI				
	i-Taukei		Indian		Fiji
	Men	Women	Men	Women	
Incidence in 2011^a^	4.03 (3.50–4.57)	4.67 (4.16–5.18)	6.48 (5.69–7.26)	7.72 (7.00–8.44)	5.02 (4.72–5.31)
Projected to 2020 from period trend only^a^	4.95 (4.36–5.54)	5.68 (5.12–6.25)	7.18 (6.35–8.01)	9.08 (8.30–9.86)	5.78 (5.46–6.10)
Projected to 2020 from period and BMI trends^b^	5.63 (4.96–6.30)	6.76 (6.13–7.40)	7.99 (7.06–8.92)	9.29 (8.44–10.14)	6.39 (6.04–6.74)
Projected: no BMI change 2011-20^b^	5.61 (4.94–6.28)	6.49 (5.87–7.11)	7.89 (6.97–8.82)	8.94 (8.11–9.77)	6.10 (5.75–6.44)
Projected from mean weight change to 2020					
Weight loss 2011–2020^b^					
−1 kg	5.52 (4.86–6.19)	6.39 (5.78–7.00)	7.78 (6.87–8.70)	8.76 (7.94–9.59)	5.93 (5.59–6.27)
−2 kg	5.45 (4.79–6.11)	6.29 (5.68–6.90)	7.65 (6.74–8.56)	8.59 (7.77–9.40)	5.77 (5.44–6.11)
−3 kg	5.45 (4.72–5.95)	6.19 (5.58–6.79)	7.53 (6.63–8.43)	8.42 (7.61–9.23)	5.62 (5.29–5.95)
−4 kg	5.30 (4.64–5.95)	6.09 (5.49–6.69)	7.40 (6.51–8.30)	8.25 (7.45–9.05)	5.46 (5.14–5.79)
Weight gain 2011–2020^b^					
+1 kg	5.68 (5.01–6.36)	6.60 (5.97–7.22)	8.05 (7.11–8.98)	9.12 (8.28–9.96)	6.27 (5.92–6.62)
+2 kg	5.76 (5.08–6.44)	6.70 (6.07–7.33)	8.18 (7.24–9.12)	9.30 (8.45–10.15)	6.44 (6.09–6.80)
+3 kg	5.84 (5.16–6.53)	6.81 (6.18–7.44)	8.32 (7.37–9.27)	9.49 (8.63–10.35)	6.62 (6.26–6.98)
+4 kg	5.93 (5.24–6.61)	6.92 (6.28–7.56)	8.46 (7.50–9.41)	9.68 (8.81–10.55)	6.81 (6.44–7.17)

^a^Incidence for 2011 and 2020 is based on applying the Styblo birth cohort method to prevalence estimates, by sex and ethnicity, age-adjusted

^b^From ethnic-specific Poisson models using survey year, sex, age, and mean BMI

If the population weight is maintained at 2011 levels, annual national T2DM incidence for 2020 is estimated as 6.1 per 1000 person-years, equivalent to a reduction of 0.3 incident cases per 1000 population in 2020 compared to projected incidence in 2020 assuming a continuation of the current-period BMI trend (6.4 per 1000 person-years). If mean population weight is reduced by 1 kg from 2011 levels, T2DM incidence in 2020 is expected to be 5.9 per 1000 person-years, equivalent to preventing 0.5 cases per 1000 in 2020 compared to the current BMI trend; and a 4-kg reduction in mean weight results in an estimated T2DM incidence of 5.5 per 1000 person-years in 2020, equivalent to a reduction of 0.9 cases per 1000 in 2020 compared to the current BMI trend. Conversely, increases of 1, 2, 3, and 4 kg in weight by 2020 are projected to increase T2DM by 0.2, 0.4, 0.5, and 0.7 per 1000 person-years by 2020, respectively, compared to weight maintenance at 2011 levels.

## Discussion

This study demonstrates an application to T2DM of a method for calculating annual incidence from sequential prevalence surveys. The method is extended to allow utilization of sequential prevalence surveys conducted over varying intervals to estimate annual T2DM incidence. Incidence is considerably more relevant than prevalence in studies of causality, has been used in disease burden estimation as a component of years lived with disability [30, 31], and is useful for estimating cases prevented by interventions.

From estimates of T2DM prevalence for 2020 based on linear trends [13], prevalence of T2DM in Fiji was predicted to be 19.3%, an increase from 15.6% in the last empirical survey (2011). By comparison, the IDF Diabetes Atlas estimate of T2DM prevalence in 2013 (10.9%) [32] was much lower, likely because they used a single survey to inform their statistical model [33], which used extensive forward and backward projections. We used six prevalence surveys to inform our modeling, which improves generalizability and limits use of extensive projection. In the present study, projections based on the effects of period, age, and linear trends in BMI, national T2DM prevalence in Fiji for 2020 is estimated to be 20.6%.

Cohort [34–36] and case-cohort [36] studies have demonstrated a positive association between T2DM incidence and weight gain. The InterAct study [36] in European populations found that for every unit standard deviation (SD) increase in BMI (4 kg/m^2^), the hazard ratio for T2DM incidence over 10 years was approximately 2.0, compared to those who did not gain weight over 10 years (average for both sexes).

Projections for Fiji imply that prevention of new T2DM cases can be achieved by reducing population average weight by relatively small amounts. The impact of such T2DM disease reduction would likely contribute to improvements in premature mortality rates and life expectancy, which have been adversely affected by increases in NCDs [37, 38]. We estimate that the rate of new cases diagnosed each year in Fijian adults in 2020 would decrease by 0.2–0.6 per 1000 person-years compared to population weight stabilization at 2011 levels. The reductions are higher when compared to the expected BMI for 2020 based on the current BMI trend increase (0.5–0.9 per 1000 person-years).

Few examples of sustained countrywide population weight loss over several years and subsequent reductions in NCDs have been documented. Most come from observational studies of countries undergoing significant economic or social changes such as Cuba [39] following the dissolution of the Soviet Union. After 1991, Cuba lost subsidies for food and fuel, which resulted in reductions in energy intake, changes in food availability (e.g., substituting vegetable oils for saturated fat), and increased active transport (e.g., walking, cycling) in place of automobiles. T2DM incidence in Cuba from diabetes registries based on primary care was stable between 1980 and 1989 (1.5–1.8 per 1000 person-years), but fell over 1990–2000 to 1.2 per 1000 person-years. Fiscal policies to encourage healthier lifestyles may be an effective tool in reducing NCD risk factors compared to health education programs alone. In the Pacific, health promotion campaigns against obesity have been operating for many years but obesity still continues to rise [13, 40]. In Tonga, animal fats and soft drinks are subject to an excise of Tonga Pa’anga (TOP) $1.00 per kg and TOP$0.50 per litre, respectively [41]. Such fiscal measures could be replicated in Fiji, but their effects have yet to be established.

The annual T2DM incidence rates in i-Taukei men (2.3 per 1000 person-years) and women (2.8 per 1000 person-years) over 1990–1994 were lower compared to Samoa (5.3, each sex) over 1991–1995 [9]; and American Samoa over 1990–1994 of (28.7, men and 21.7, women) – for comparable age groups [9]. The lower annual incidence in Fiji i-Taukei compared to Samoans and American Samoans may be related to lower levels of obesity in Fiji [12] compared to Samoa and American Samoa. Between 1980 and 2011, obesity prevalence (BMI ≥30 kg/m^2^) increased at a rate of 3.0% per five years in i-Taukei men (from 12.6 to 28.9%) and 3.8% per five years in i-Taukei women (30.1–52.9%) [13]. For a similar period (1978–2013), the rate of obesity increase in Samoans was 3.6% per five years in men (from 23.5 to 53.1%), and 4.6% per five years in women (43.7 to 73.4%) [42], with higher baseline obesity prevalence in Samoans also indicating a longer duration of obesity than in Fiji. Both magnitude and duration of obesity have been shown to affect increases in T2DM incidence, which has been demonstrated in Pima Indians [43], American men and women [34, 35], British men [44], and Japanese men [45]. Relatively high T2DM incidence rates have been reported in other small island states such as Mauritius, where estimated incidence averaged 21.0 per 1000 person-years over 1987–1998 [2].

The method used to estimate T2DM incidence in this study circumvents numerous pitfalls compared to traditional methods of calculating T2DM incidence, including cohort studies affected by low generalizability and/or attrition bias, under-enumeration of cases of T2DM not included on pharmaceutical registers, or on self-report of diagnosed T2DM within a reference period from cross-sectional surveys. To the authors’ knowledge, the present study is the first to apply Styblo’s birth cohort incidence calculation method to estimate T2DM incidence. From a statistical perspective, the main drawback encountered using the method was underdispersion in subsequent Poisson regression modeling of factors associated with T2DM incidence.

## Conclusion

T2DM incidence has increased in all population groups in Fiji, and the increase is expected to continue into the near future. This study demonstrates a novel method of calculating incidence from already available data from previous sequential population-based prevalence surveys, with projections based on various population weight change scenarios, which may be replicated in other countries. Estimates and projections of incidence of T2DM and its accumulated risk over an adult lifetime provide policymakers with indicators of the expected burden of new cases of T2DM in Fiji and highlight population groups most at risk of the disease.

## Additional file

Additional file 1: Supplement 1: Method for deriving estimates of T2DM incidence; and Supplement 2: Age-specific T2DM prevalence by ethnicity and sex. (DOCX 331 kb)

## Abbreviations

BMI: Body mass index
FBG: Fasting whole blood glucose
FPG: Fasting plasma glucose
NCD: Non-communicable disease
NCVDS: National Cardiovascular Disease and Diabetes Survey
NNS: National Nutrition Survey
SD: Standard deviation
T2DM: Type 2 diabetes mellitus
TOP: Tongan Pa’anga

## References

[1] Soderberg S, Zimmet P, Tuomilehto J, de Courten M, Dowse GK, Chitson P (2004). High incidence of type 2 diabetes and increasing conversion rates from impaired fasting glucose and impaired glucose tolerance to diabetes in Mauritius. J Intern Med.

[2] McCambridge J, Witton J, Elbourne DR (2014). Systematic review of the Hawthorne effect: new concepts are needed to study research participation effects. J Clin Epidemiol.

[3] Geiss LS, Pan L, Cadwell B, Gregg EW, Benjamin SM, Engelgau MM (2006). Changes in incidence of diabetes in U.S. adults, 1997–2003. Am J Prev Med.

[4] Monesi L, Baviera M, Marzona I, Avanzini F, Monesi G, Nobili A (2012). Prevalence, incidence and mortality of diagnosed diabetes: evidence from an Italian population-based study. Diabet Med.

[5] AIHW Australian Institute of Health and Welfare (2009). Insulin-treated treated diabetes in Australia 2000-2007. Diabetes series no. 11. Cat. No. CVD 45.

[6] Stovring H, Andersen M, Beck-Nielsen H, Green A, Vach W (2003). Rising prevalence of diabetes: evidence from a Danish pharmacoepidemiological database. Lancet.

[7] An Integrative Metaregression Framework for Descriptive Epidemiology. Edited by Flaxman AD, Vos T, Murray CJL. Seattle: University of Washington Press, 2015.

[8] McGarvey ST (2001). Cardiovascular disease (CVD) risk factors in Samoa and American Samoa, 1990-1995. Pac Health Dialog.

[9] Dowse GK, Zimmet PZ, Finch CF, Collins VR (1991). Decline in incidence of epidemic glucose intolerance in Nauruans: implications for the ‘thrifty genotype’. Am J Epidemiol.

[10] Samoa Bureau of Statistics (1992). Population and housing census 1991.

[11] Rakaseta VL (1999). American Samoa population profile: a guide for planners and policy-makers.

[12] Lin S, Tukana I, Linhart C, Morrell S, Taylor R, Vatucawaqa P (2016). Diabetes and obesity trends in Fiji over 30 years. J Diabetes.

[13] Fiji Bureau of Statistics. 2007 Census of Population and Housing. Suva: Government of Fiji; 2007.

[14] Stýblo K, Meijer J, Sutherland I (1969). Tuberculosis Surveillance Research Unit Report No. 1: the transmission of tubercle bacilli; its trend in a human population. Bull Int Union Tuberc.

[15] Zimmet P, Taylor R, Ram P, King H, Sloman G, Raper LR (1983). Prevalence of diabetes and impaired glucose tolerance in the biracial (Melanesian and Indian) population of Fiji: a rural-urban comparison. Am J Epidemiol.

[16] NFNC (1995). 1993 National Nutrition Survey: Main Report.

[17] WHO (2007). Fiji Non-communicable Diseases STEPswise Risk Factor (NCD STEPS) Survey 2002.

[18] NFNC (2004). Fiji National Nutrition Survey: Main Report.

[19] Langley D (1953). Dietary surveys and growth records in a Fijian village, Naduri.

[20] Wilkins RM (1963). Dietary survey in a Fijian village, Naduri, Nadroga.

[21] Tuivaga J, Seniloli S (1996). Report of the fifth decennial Naduri Nutrition and Health Survey.

[22] Cassidy JT (1967). Diabetes in Fiji. NZ Med J.

[23] Russell-Jones DL, Hoskins E, Kearney R, Morris R, Katoaga S, Slavin B (1990). Rural/urban differences of diabetes - impaired glucose tolerance, hypertension, obesity, glycosylated haemoglobin, nutritional proteins, fasting cholesterol and apolipoproteins in Fijian Melanesians over 40. Q J Med.

[24] Hoskins PL, Handelsman DJ, Hannelly T, Silink M, Yue DK, Turtle JR (1987). Diabetes in the Melanesian and Indian peoples of Fiji: a study of risk factors. Diabetes Res Clin Pract.

[25] Hoskins PL, Handelsman DJ, Hannelly T, Silink M, Yue DK, Turtle JR (1987). Glycosylated haemoglobin as an index of the prevalence and severity of diabetes in biethnic Fiji. Diabetes Res Clin Pract.

[26] Brian G, Ramke J, Maher L, Page A, Szetu J (2010). The prevalence of diabetes among adults aged 40 years and over in Fiji. NZ Med J.

[27] WHO (2006). Definition and diagnosis of diabetes mellitus and intermediate hyperglycemia: report of a WHO/IDF consultation.

[28] Armitage P, Berry G, Matthews JNS (2001). Statistical Methods in Medical Research.

[29] Day NE (1992). Cancer Incidence in Five Continents. Cumulative rate and cumulative risk. IARC Sci Publ.

[30] World Health Organisation (WHO), International Association for the Study of Obesity (IASO), International Obesity Task Force (IOTF) (2000). The Asia-Pacific Perspective: Redefining obesity and its treatment.

[31] Vos T, Flaxman AD, Naghavi M, Lozano R, Michaud C, Ezzati M (2012). Years lived with disability (YLDs) for 1160 sequelae of 289 diseases and injuries 1990-2010: a systematic analysis for the Global Burden of Disease Study 2010. Lancet.

[32] International Diabetes Federation (2013). IDF diabetes atlas.

[33] Guariguata L, Whiting DR, Hambleton I, Beagley J, Linnenkamp U, Shaw JE (2014). Global estimates of diabetes prevalence for 2013 and projections for 2035. Diabetes Res Clin Pract.

[34] Hu FB, Manson JE, Stampfer MJ, Colditz G, Liu S, Solomon CG (2001). Diet, lifestyle, and the risk of type 2 diabetes mellitus in women. N Engl J Med.

[35] Narayan KMV, Boyle JP, Thompson TJ, Gregg EW, Williamson DF (2007). Effect of BMI on lifetime risk for diabetes in the U.S. Diabetes Care.

[36] The InterAct Consortium (2012). Long-term risk of incident type 2 diabetes and measures of overall and regional obesity: the EPIC-InterAct Case-Cohort Study. PLoS Med.

[37] Carter K, Cornelius M, Taylor R, Ali SS, Rao C, Lopez AD (2011). Mortality trends in Fiji. Aust NZ J Public Health.

[38] Taylor R, Carter K, Naidu S, Linhart C, Azim S, Rao C (2013). Divergent mortality trends by ethnicity in Fiji. A NZ J Public Health.

[39] Franco M, Bilal U, Orduñez P, Benet M, Morejon A, Caballero B (2013). Population-wide weight loss and regain in relation to diabetes burden and cardiovascular mortality in Cuba 1980-2010: repeated cross sectional surveys and ecological comparison of secular trends. BMJ.

[40] Thow AM, Snowdon W, Schultz JT, Leeder S, Vivili P, Swinburn BA (2011). The role of policy in improving diets: experiences from the Pacific Obesity Prevention in Communities food policy project. Obes Rev.

[41] Government of Tonga (2013). Excise Tax (Amendment) (No.2) Order 2013.

[42] Lin S, Naseri T, Linhart C, Morrell S, Taylor R, McGarvey ST (2016). Trends in diabetes and obesity in Samoa over 35 years, 1978-2013. Diabetic Med.

[43] Everhart JE, Pettitt DJ, Bennett PH, Knowler WC (1992). Duration of obesity increases the incidence of NIDDM. Diabetes.

[44] Wannamethee SG, Shaper AG (1999). Weight change and duration of overweight and obesity in the incidence of type 2 diabetes. Diabetes Care.

[45] Sakurai Y (2000). Duration of obesity and risk of non-insulin-dependent diabetes mellitus. Biomed Pharmacother.

