# Supplementary material for: Diabetes incidence and projections from prevalence surveys in Fiji
Source: Popul Health Metr. 2016 Nov 25;14:45. doi: 10.1186/s12963-016-0114-0 (PMC5124232; doi:10.1186/s12963-016-0114-0)
Supplement: Additional file 1: — Supplement 1: Method for deriving estimates of T2DM incidence; and Supplement 2: Age-specific T2DM prevalence by ethnicity and sex. (DOCX 331 kb) [file 12963_2016_114_MOESM1_ESM.docx]

**Diabetes incidence from prevalence surveys in Fiji and projections with various obesity scenarios**

*S MORRELL, S LIN, I TUKANA, C LINHART, R TAYLOR, P VATUCAWAQA, DJ MAGLIANO, P ZIMMET*

**SUPPLEMENT S1: Method for deriving estimates of type 2 diabetes mellitus (T2DM) incidence**

Annual T2DM incidence was estimated by adapting the method developed by Styblo [1] to estimate tuberculosis incidence from tuberculosis prevalence.

The steps in the approach are as follows:

1. Prevalence (*Q_a,b_*) of a disease for a given age (*a*) and year of birth (*b*), is 1- the cumulated probability of not acquiring the disease in any year from year of birth *b* to age *a*, given as:

where *p_b_* is the probability of acquiring the disease in the year of birth, *p_b+1_* the probability of acquiring the disease in the following year, and so on by age = *a.*

This is written more conveniently as:

 where,

*q_ab_ (≡1-p_ab_)* is the probability of not acquiring the disease in a given year

Thus,

For time as a continuous variable, from birth *b* to age *a* for example,


2. For each age (*a*) and year of birth (*b*), the average annual probability of becoming a case of disease up to that age (*p_b_*) is assumed to increase more or less smoothly. That is, for a given age *a*, *q* is a constant and *Q_a,b_* becomes:

Thus, *Q_a,b_ = q^a^*

=> *q = (Q_a,b_)^1/a^*, the *‘a’*th root of *Q_a,b_*

3. For each age separately, a linear regression estimate is made from the earliest to the last year of birth for each of the cohorts at that age, *q* is modeled as a function of year of birth *b*. Styblo *et al*. found that the best linear function of *q(t)*, suitable for a linear regression fit of year of birth, was *log(-logq(t)).*That is,

*log(-log(q(t)) = c + st*

*=> -logq(t) = e^c+st^* ......(2)

Substituting this into (1) above, the formula for *logQ_a,b_* gives:

This is easily integrable, giving:

Substituting

gives:

For *t = b + a*, the age of the person at the time of measurement, the log of the risk of not acquiring the disease by age *a* becomes:

......(3)

When *logq(b+a)* is exponentiated and then subtracted from 1, the result is the estimate of the probability of acquiring the disease at age *a* at the time (year) of measurement (≡ *p(b+a)*), the incidence for that year.

4. As the intervals between the surveys used in the present study were not evenly spaced, age-specific T2DM prevalences were interpolated between the survey years using linear regression to produce estimates of annual age-specific prevalences of T2DM for each sex and ethnic group. The quantity *s*, the regression estimate for the time trend in diabetes risk for a given age, was estimated from each survey by linear regression modeling of age-specific T2DM prevalence at each survey against year of birth. Age was taken as the median age for each five-year age group, and the year of birth for each age calculated by subtracting the median age from the year of the survey.

5. Annual age-specific incidence estimates for each sex and ethnic group were then age-standardized to the Fiji census 25-64 year population of 2007 separately by sex and ethnicity, to produce four directly age-standardized 25-64 incidence trends for 1980–2010.

**SUPPLEMENT S2: Age-specific T2DM prevalence (%) for included surveys by ethnicity and sex**

**Table 1.** Diabetes (T2DM) prevalence (%) for Fiji adults aged 25-64 years by ethnic-specific sex groups for each survey

|  | 1980 NCVDS | 1993 NNS | 2002 STEPS | 2004 NNS | 2011 STEPS |
| --- | --- | --- | --- | --- | --- |
| **i-Taukei men** | |  |  |  |  |
| 25-29 | 2.9 | 2.5 | 4.9 | 3.2 | 2.9 |
| 30-34 | 1.3 | 3.8 | 2.9 | 4.8 | 6.8 |
| 35-39 | 0.9 | 4.8 | 3.9 | 6.4 | 5.8 |
| 40-44 | 2.5 | 7.5 | 4.0 | 7.9 | 9.7 |
| 45-49 | 6.3 | 8.1 | 9.9 | 14.7 | 7.1 |
| 50-54 | 4.6 | 11.9 | 17.4 | 16.4 | 24.7 |
| 55-59 | 5.6 | 13.6 | 19.4 | 22.0 | 24.2 |
| 60-64 | 6.4 | 17.4 | 24.5 | 27.8 | 32.2 |
| **25-64†** | **3.2** | **5.3** | **8.5** | **8.6** | **11.1** |
| 95% CI | 3.0-3.5 | 4.8-5.8 | 7.8-9.1 | 8.0-9.1 | 10.3-11.9 |
| **i-Taukei women** | |  |  |  |  |
| 25-29 | 3.0 | 2.0 | 5.9 | 3.0 | 3.4 |
| 30-34 | 2.5 | 3.5 | 5.4 | 3.9 | 9.5 |
| 35-39 | 4.9 | 4.4 | 2.6 | 5.9 | 1 |
| 40-44 | 3.6 | 6.5 | 14.9 | 9.0 | 17.4 |
| 45-49 | 7.1 | 10.0 | 19.2 | 11.5 | 15.6 |
| 50-54 | 11.2 | 9.9 | 26.1 | 19.1 | 26.3 |
| 55-59 | 12.1 | 18.1 | 31.1 | 24.6 | 27.3 |
| 60-64 | 11.4 | 20.7 | 23.9 | 27.7 | 27.5 |
| **25-64†** | **5.3** | **9.5** | **12.8** | **11.1** | **13.6** |
| 95% CI | 4.9-5.8 | 8.7-10.3 | 12.0-13.6 | 10.4-11.7 | 12.7-14.5 |
| **Indian men** | |  |  |  |  |
| 25-29 | 0.6 | 5.3 | 8.5 | 6.2 | 2.1 |
| 30-34 | 6.6 | 8.4 | 5.1 | 8.6 | 9.8 |
| 35-39 | 5.5 | 10.4 | 26.0 | 12.5 | 6.5 |
| 40-44 | 15.5 | 12.5 | 26.4 | 16.9 | 27.1 |
| 45-49 | 16.9 | 23.4 | 35.6 | 23.6 | 26.6 |
| 50-54 | 32.9 | 34.5 | 40.2 | 31.1 | 30 |
| 55-59 | 16.6 | 39.1 | 39.1 | 47.5 | 31.2 |
| 60-64 | 44.0 | 41.3 | 54.9 | 40.0 | 44.1 |
| **25-64†** | 11.1 | **17.5** | **25.4** | **21.0** | **17.9** |
| 95% CI | 10.2-12.0 | 16.2-18.8 | 23.4-27.4 | 19.7-22.2 | 16.4-19.4 |
| **Indian women** | |  |  |  |  |
| 25-29 | 1.1 | 4.2 | 5.3 | 6.1 | 6.2 |
| 30-34 | 11.0 | 6.0 | 7.7 | 7.1 | 9 |
| 35-39 | 9.0 | 11.6 | 12.2 | 12.2 | 15.3 |
| 40-44 | 11.7 | 16.2 | 16.4 | 16.0 | 23.6 |
| 45-49 | 15.5 | 22.8 | 18.7 | 28.9 | 29.2 |
| 50-54 | 23.8 | 21.5 | 36.4 | 29.4 | 29.1 |
| 55-59 | 22.9 | 37.2 | 49.9 | 38.8 | 36.2 |
| 60-64 | 16.4 | 29.9 | 46.6 | 55.5 | 38.1 |
| **25-64†** | **11.2** | **12.8** | **20.2** | **17.4** | **19.9** |
| 95% CI | 10.3-12.1 | 11.7-13.8 | 18.9-21.4 | 16.4-18.5 | 18.6-21.3 |

† Age-standardized to nearest previous census. Prevalence was calculated after adjusting to nearest previous census for urban-rural.

**Table 2.** Annual diabetes (T2DM) incidence (per 1000) for Fiji adults 25-64 years by ethnic-specific sex groups

|  | 1980 | 1985 | 1990 | 1995 | 2000 | 2005 | 2010 | 2015 | 2020 |
| --- | --- | --- | --- | --- | --- | --- | --- | --- | --- |
| **i-Taukei men** | | | | | | | | | |
| 25-29 | 1.20 | 1.22 | 1.24 | 1.26 | 1.27 | 1.29 | 1.31 | 1.33 | 1.35 |
| 30-34 | 0.69 | 0.92 | 1.17 | 1.41 | 1.65 | 1.89 | 2.14 | 2.39 | 2.64 |
| 35-39 | 0.72 | 1.00 | 1.29 | 1.58 | 1.86 | 2.15 | 2.45 | 2.74 | 3.04 |
| 40-44 | 0.91 | 1.37 | 1.82 | 2.29 | 2.76 | 3.23 | 3.72 | 4.20 | 4.70 |
| 45-49 | 1.80 | 1.92 | 2.05 | 2.18 | 2.30 | 2.43 | 2.56 | 2.69 | 2.82 |
| 50-54 | 1.61 | 2.61 | 3.64 | 4.71 | 5.80 | 6.94 | 8.11 | 9.33 | 10.59 |
| 55-59 | 1.75 | 2.83 | 3.95 | 5.11 | 6.31 | 7.56 | 8.86 | 10.21 | 11.63 |
| 60-64 | 2.23 | 3.54 | 4.92 | 6.35 | 7.85 | 9.43 | 11.09 | 12.83 | 14.69 |
| **25-64†** | **1.22** | **1.64** | **2.08** | **2.52** | **2.98** | **3.45** | **3.93** | **4.43** | **4.95** |
| 95% CI | 1.13-1.31 | 1.52-1.77 | 1.92-2.23 | 2.36-2.68 | 2.82-3.14 | 3.27-3.63 | 3.73-4.13 | 4.22-4.64 | 4.74-5.16 |
| **i-Taukei women** | | | | | | | | | |
| 25-29 | 1.22 | 1.27 | 1.33 | 1.39 | 1.45 | 1.50 | 1.56 | 1.62 | 1.68 |
| 30-34 | 0.16 | 0.82 | 1.49 | 2.18 | 2.87 | 3.57 | 4.28 | 5.01 | 5.75 |
| 35-39 | 1.22 | 1.14 | 1.06 | 0.98 | 0.91 | 0.83 | 0.75 | 0.67 | 0.59 |
| 40-44 | 0.93 | 1.78 | 2.64 | 3.53 | 4.43 | 5.36 | 6.31 | 7.28 | 8.28 |
| 45-49 | 2.16 | 2.66 | 3.17 | 3.68 | 4.20 | 4.74 | 5.28 | 5.83 | 6.40 |
| 50-54 | 2.64 | 3.42 | 4.22 | 5.04 | 5.88 | 6.76 | 7.66 | 8.59 | 9.55 |
| 55-59 | 3.30 | 4.15 | 5.03 | 5.93 | 6.88 | 7.85 | 8.87 | 9.92 | 11.02 |
| 60-64 | 3.44 | 4.25 | 5.09 | 5.95 | 6.84 | 7.77 | 8.73 | 9.73 | 10.77 |
| **25-64†** | **1.53** | **2.00** | **2.49** | **2.99** | **3.50** | **4.02** | **4.56** | **5.12** | **5.68** |
| 95% CI | 1.41-1.64 | 1.86-2.15 | 2.32-2.66 | 2.83-3.15 | 3.35-3.65 | 3.85-4.19 | 4.38-4.75 | 4.93-5.30 | 5.50-5.87 |
| **Indian men** | | | | | | | | | |
| 25-29 | 1.27 | 1.61 | 1.96 | 2.32 | 2.67 | 3.03 | 3.39 | 3.75 | 4.12 |
| 30-34 | 2.19 | 2.28 | 2.36 | 2.44 | 2.52 | 2.60 | 2.69 | 2.77 | 2.85 |
| 35-39 | 3.07 | 3.38 | 3.70 | 4.02 | 4.34 | 4.66 | 4.99 | 5.32 | 5.66 |
| 40-44 | 3.94 | 4.52 | 5.11 | 5.71 | 6.32 | 6.95 | 7.60 | 8.25 | 8.93 |
| 45-49 | 5.45 | 6.00 | 6.56 | 7.13 | 7.72 | 8.32 | 8.93 | 9.56 | 10.21 |
| 50-54 | 8.02 | 7.95 | 7.88 | 7.81 | 7.75 | 7.68 | 7.61 | 7.54 | 7.48 |
| 55-59 | 6.97 | 7.81 | 8.68 | 9.59 | 10.52 | 11.50 | 12.52 | 13.59 | 14.70 |
| 60-64 | 9.57 | 9.68 | 9.79 | 9.91 | 10.02 | 10.14 | 10.25 | 10.37 | 10.49 |
| **25-64†** | **4.24** | **4.58** | **4.93** | **5.29** | **5.65** | **6.02** | **6.40** | **6.79** | **7.18** |
| 95% CI | 4.02-4.46 | 4.36-4.80 | 4.71-5.15 | 5.07-5.50 | 5.43-5.86 | 5.80-6.24 | 6.17-6.62 | 6.57-7.00 | 6.98-7.38 |
| **Indian women** | | | | | | | | | |
| 25-29 | 0.79 | 1.22 | 1.66 | 2.10 | 2.55 | 3.00 | 3.45 | 3.91 | 4.37 |
| 30-34 | 3.15 | 3.11 | 3.07 | 3.02 | 2.98 | 2.94 | 2.90 | 2.85 | 2.81 |
| 35-39 | 3.07 | 3.45 | 3.83 | 4.22 | 4.61 | 5.01 | 5.41 | 5.82 | 6.23 |
| 40-44 | 3.39 | 3.83 | 4.27 | 4.72 | 5.17 | 5.63 | 6.10 | 6.58 | 7.06 |
| 45-49 | 5.22 | 6.08 | 6.97 | 7.89 | 8.84 | 9.82 | 10.85 | 11.90 | 13.01 |
| 50-54 | 5.87 | 6.27 | 6.67 | 7.08 | 7.50 | 7.92 | 8.36 | 8.80 | 9.25 |
| 55-59 | 6.96 | 8.12 | 9.33 | 10.60 | 11.95 | 13.37 | 14.88 | 16.50 | 18.23 |
| 60-64 | 5.61 | 7.40 | 9.32 | 11.38 | 13.61 | 16.03 | 18.69 | 21.63 | 24.92 |
| **25-64†** | **3.84** | **4.40** | **4.99** | **5.60** | **6.23** | **6.89** | **7.58** | **8.31** | **9.08** |
| 95% CI | 3.63-4.04 | 4.19-4.62 | 4.77-5.21 | 5.40-5.80 | 6.05-6.40 | 6.72-7.06 | 7.43-7.73 | 8.19-8.42 | 9.01-9.15 |

† Age-standardized to nearest previous census.

**SUPPLEMENT S3: Use of Poisson regression to model under-dispersed data**

T2DM prevalence based on ethnic-specific regression models yielded results with p-values of 0.022 when the deviance was scaled (model deviance/degrees of freedom) due to significant under-dispersion. P-values for corresponding incidence models with scaled deviance were 0.047 for Indians and 0.055 for Fiji overall. In these analyses, Conway-Maxwell Poisson regression models that are normally used for modeling under-dispersed count data failed to converge.

Under-dispersion occurs when the model variance is considerably less than the mean value being modeled, violating the Poisson assumption of equality of variance and mean, as also occurs with over-dispersed models. Since the Conway-Maxwell Poisson models [2] failed to converge, the less satisfactory measure of scaling the deviance was used to correct parameter standard error estimates for under-dispersion in Poisson regression.

**SUPPLEMENT REFERENCES**

1. [Stýblo K](http://www.ncbi.nlm.nih.gov/pubmed/?term=St%C3%BDblo%20K%5BAuthor%5D&cauthor=true&cauthor_uid=5004883), [Meijer J](http://www.ncbi.nlm.nih.gov/pubmed/?term=Meijer%20J%5BAuthor%5D&cauthor=true&cauthor_uid=5004883), [Sutherland I](http://www.ncbi.nlm.nih.gov/pubmed/?term=Sutherland%20I%5BAuthor%5D&cauthor=true&cauthor_uid=5004883). Tuberculosis Surveillance Research Unit Report No. 1: the transmission of tubercle bacilli; its trend in a human population. [*Bull Int Union Tuberc*](http://www.ncbi.nlm.nih.gov/pubmed/5004883) 1969; 42:1-104.
2. Shmueli G, Minka TP, Kadane JB, Borle S, Boatwright P. A useful distribution for fitting discrete data: revival of the Conway–Maxwell–Poisson distribution. J R Stat Soc: Series C (Applied Statistics) 2005; 54(1):127–142.
